# Characterization of spliced leader *trans-*splicing in a photosynthetic rhizarian amoeba, *Paulinella micropora*, and its possible role in functional gene transfer

**DOI:** 10.1371/journal.pone.0200961

**Published:** 2018-07-19

**Authors:** Mitsuhiro Matsuo, Atsushi Katahata, Soichirou Satoh, Motomichi Matsuzaki, Mami Nomura, Ken-ichiro Ishida, Yuji Inagaki, Junichi Obokata

**Affiliations:** 1 Graduate School of Life and Environmental Sciences, Kyoto Prefectural University, Kyoto, Japan; 2 Department of Biomedical Chemistry, Graduate School of Medicine, The University of Tokyo, Tokyo, Japan; 3 Graduate School of Life and Environmental Sciences, University of Tsukuba, Tsukuba, Japan; 4 Center for Computational Sciences, University of Tsukuba, Tsukuba, Japan; University of Cambridge, UNITED KINGDOM

## Abstract

*Paulinella micropora* is a rhizarian thecate amoeba, belonging to a photosynthetic *Paulinella* species group that has a unique organelle termed chromatophore, whose cyanobacterial origin is distinct from that of plant and algal chloroplasts. Because acquisition of the chromatophore was quite a recent event compared with that of the chloroplast ancestor, the *Paulinella* species are thought to be model organisms for studying the early process of primary endosymbiosis. To obtain insight into how endosymbiotically transferred genes acquire expression competence in the host nucleus, here we analyzed the 5′ end sequences of the mRNAs of *P*. *micropora* MYN1 strain with the aid of a cap-trapper cDNA library. As a result, we found that mRNAs of 27 genes, including endosymbiotically transferred genes, possessed the common 5′ end sequence of 28–33 bases that were posttranscriptionally added by spliced leader (SL) *trans*-splicing. We also found two subtypes of SL RNA genes encoded by the *P*. *micropora* MYN1 genome. Differing from the other SL *trans*-splicing organisms that usually possess poly(A)-less SL RNAs, this amoeba has polyadenylated SL RNAs. In this study, we characterize the SL *trans*-splicing of this unique organism and discuss the putative merits of SL *trans*-splicing in functional gene transfer and genome evolution.

## Introduction

Splice leader (SL) *trans*-splicing is an RNA maturation process that adds a short leader sequence of 16–52 bases to the 5′ end of precursor mRNAs [1]. This process was first reported for *Trypanosoma* [2, 3] and later found in diverse organisms [1, 4]. SL *trans*-splicing occurs similarly to *cis*-splicing [5] and is mediated by the *trans*-spliceosome that contains U2, U4, U5, U6 snRNPs and SL snRNP, which is in place of U1 snRNP that is used for *cis*-spicing [6–11]. SL snRNP carries SL RNA to the 5′ end of the recipient precursor mRNAs [5, 12].

Physiological roles of the SL *trans*-splicing were intensively studied in trypanosomes and nematodes, revealing that it is essential to their viability [13, 14]. These organisms possess polycistronic gene clusters whose transcripts are converted to monocistronic mature mRNAs by SL *trans*-splicing [15–18]. SL *trans*-splicing can replace their unfavorable 5′ UTR sequences for translation with preferable ones [19–22].

One of the peculiarities of SL *trans*-splicing is its sporadic appearance in eukaryotic lineages. SL *trans*-splicing was reported in metazoan [23], Euglenozoa [2, 3, 24], dinoflagellates [25], Perkinsea [26], and Rhizaria [27] but not found in fungi, plants and most other protists [1]. In metazoan, SL *trans*-splicing is also patchily distributed; for example, tunicates in chordates [28, 29] and copepod and amphipod [23, 30] in arthropoda perform SL *trans*-splicing, but no evidence has been found in closely related vertebrates and insects [1]. The fragmented distribution should reflect multiple gains or losses of the SL *trans*-splicing system in the eukaryotic lineage [31], and repeated acquisitions of SL *trans*-splicing are suggested in metazoan evolution [23, 32]. Interestingly, from the observation that an U1 snRNA fragment can be converted into SL RNA by addition of only splice donor sequence and a few mutations [33], it is proposed that SL *trans*-splicing might have emerged by mutation of U snRNAs [1, 23]. SL *trans*-splicing might have easily emerged in eukaryote evolution.

Photosynthetic *Paulinella* species are rhizarian thecate amoebas with a unique photosynthetic organelle, namely, chromatophora, derived from the cyanobacterial ancestor distinct from that of plant and algal chloroplasts [34–36]. Its endosymbiosis is thought to have occurred about 100 million years ago [37]; hence, its chromatophore is a very young organelle compared with circa 1.9 billion-year-old chloroplasts [38]. Therefore, *Paulinella* species attracted the attention of researchers interested in the early process of primary endosymbiosis and endosymbiotic gene transfer (EGT) events [39, 40]. For these purposes, genome and transcriptome analyses [27] have been performed for the CCAC0185 strain of *Paulinella chromatophora* [36].

We have been interested in the molecular mechanisms of EGT and HGT (horizontal gene transfer) especially in the process of how transferred alien genes obtain expression competence in their host nucleus. In this respect, the promoter and 5′ UTR of the recently transferred genes may contain rich information. In this study, we analyzed the 5′ UTRs of mRNAs in the *P*. *micropora* MYN1 strain, a member of the photosynthetic *Paulinella* group [41] but phylogenetically distant from *P*. *chromatophora* [42]. We found that the nuclear genes of *P*. *micropora* MYN1, including those derived from EGT, are also at least in part subjected to SL *trans-*splicing, as implicated in *P*. *chromatophora* [27]. This study shows detailed characterization of SL *trans-*splicing of *P*. *micropora*, and we also discuss the potential merits of SL *trans*-splicing from the viewpoint of functional gene transfer and genome evolution.

## Results

### Detection of SL *trans*-splicing in *P*. *micropora* MYN1

To obtain information about the 5′ end sequences of *P*. *micropora* MYN1’s transcripts, we sequenced the cap-trapper cDNA libraries [43] of this organism using the Ion PGM sequencer (Thermo Fisher Scientific, Waltham, MA, USA). After trimming the linker- and adapter-sequences, the reads of organelle transcripts and rRNAs were discarded. Finally, we obtained 325,863 reads (44 Mb) with the average read length of 136 bases. Mapping of these reads on the actin and polyubiquitin mRNAs ensured that the 5′ end sequences were enriched (S1 Fig). Because axenic culture has never been established for *P*. *micropora*, the sequence reads should include those from the contaminating bacteria. Therefore, we removed the reads that were highly similar to bacterial genomes. Furthermore, we collected the reads that encode N-terminal 50 amino acid residues of 606 independent eukaryotic proteins in the public database (NCBI-nr) by BLASTX (e-value < 1e-5) to obtain a more reliable dataset, and subjected them to the following analyses.

**Fig 1:**
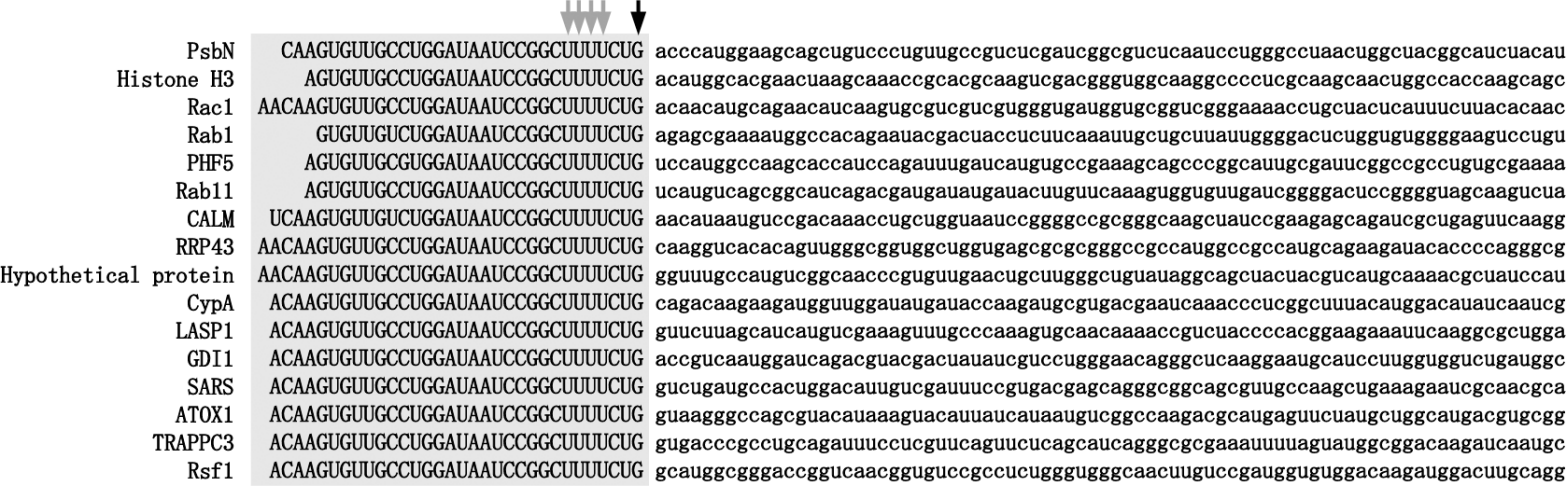
Alignment of the spliced leader *trans*-spliced mRNAs of *P*. *micropora*. cDNAs contain 28–33 base common sequences at their 5′ ends. These contigs were annotated using COG and NCBI-nr database (PsbN; photosystem II PsbN, Histone H3, Rac1; Ras-related protein Rac1, Rab1; GTP-binding protein, PHF5; PHF5-domain-containing protein, Rab11; GTPase Rab11/YPT3, CALM; Calmodulin, RRP43; Exosome complex exonuclease RRP43, CypA; Cyclophilin, LASP1; LIM and SH3 domain protein, GDI1; Rab GDP dissociation inhibitor alpha, SARS; seryl-tRNA synthetase, ATOX1; copper transport protein ATOX1, TRAPPC3; trafficking protein particle complex subunit 3, Rsf1; RNA-binding protein Rsf1). The common sequence is shaded, with the UUU triplet and 3′ terminal G indicated by the gray and black arrows, respectively.

The bioinformatics analysis of the obtained reads revealed that 16 distinct genes, including a *psbN* gene [40], contain a common 28–33 base sequence at their 5′ ends (Fig 1). This common sequence was confirmed by RT-PCR and Sanger sequencing of 8 genes, indicating that they are not sequencing artifacts.

It is known that the existence of 15–52 base common sequences at the 5′ ends of various mRNAs is a characteristic of SL *trans*-splicing. Furthermore, the 5′ end common sequences of *P*. *micropora* contain UUU triplet and had G nucleotide at the 3′ terminus (Fig 1), which are typical features of SL sequences shared by SL *trans*-splicing organisms known thus far [4].

**Fig 2:**
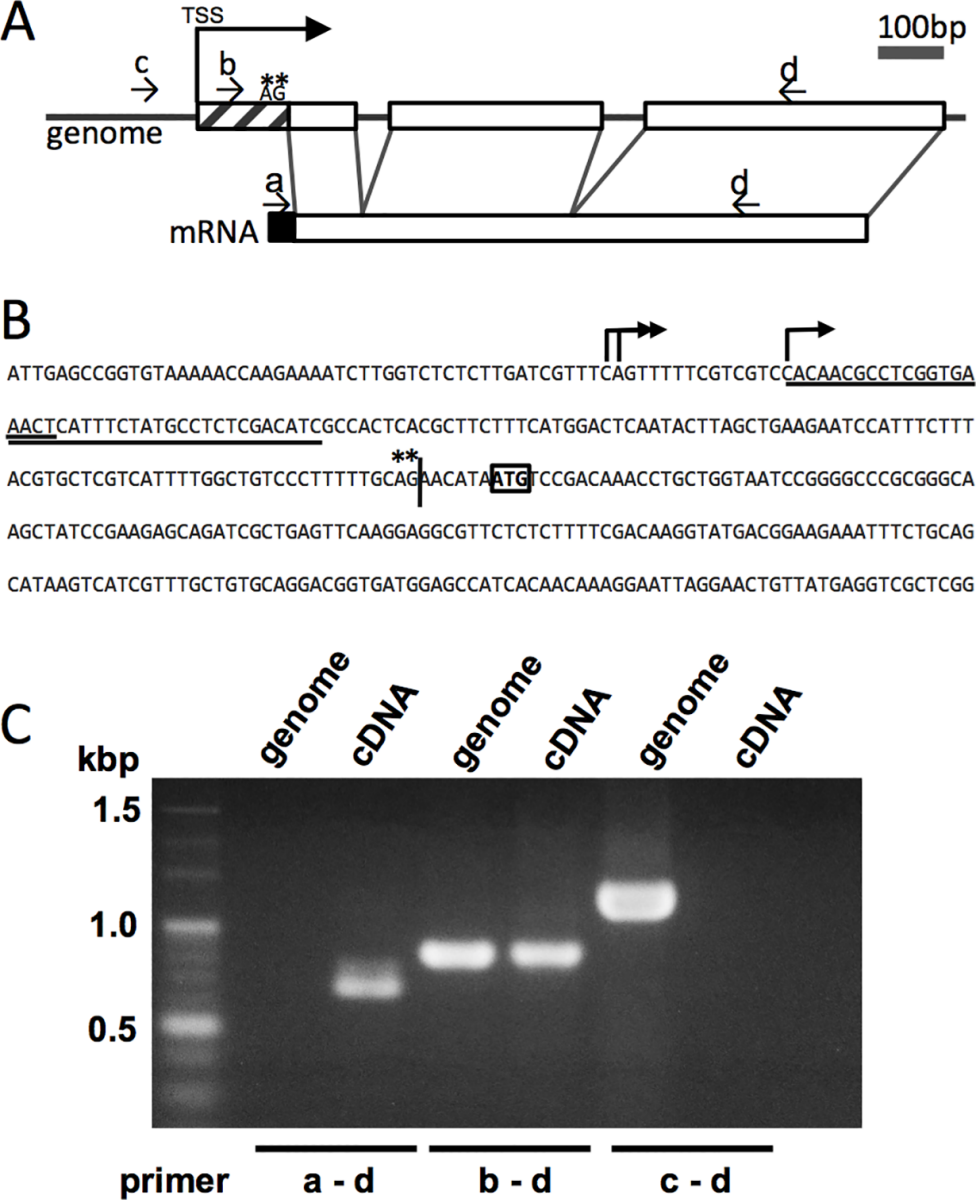
Structure of the calmodulin gene of *P*. *micropora*. **A.** Schematic model of the calmodulin gene and its mRNA. Primers used for PCR- and RT-PCR analyses are shown by small arrows a, b, c, and d. The outron and spliced leader are shown as hashed and black boxes, respectively. The thick arrow indicates the transcription start site (TSS). **B.** Genomic sequence of the calmodulin gene, with the ATG initiation codon boxed. The junction of the outron and exon is indicated by the vertical bar. TSS and the pyrimidine rich region are indicated by the thick arrow and dots, respectively. The underlines show the position of primer b (consisting of two nested primers). **C.** PCR profiles of the calmodulin gene using genomic and cDNA templates.

To confirm that SL *trans*-splicing occurs in *P*. *micropora*, we analyzed an SL *trans*-spliced gene that encodes calmodulin (Fig 2). We cloned a calmodulin gene from –588 to +758 relative to ATG initiation codon and found that it contained two introns. SL sequence was not found in the genomic sequence, and instead a 3′ acceptor splicing motif, i.e., AG (Fig 2A and 2B, asterisk) was found at the predicted position. Structural organization of the genomic and cDNA sequences was compared using the PCR primers shown in Fig 2A (arrows a, b, c, and d). A primer pair a–d amplified a fragment only from cDNA but not from genomic DNA, indicating that the SL sequence was added to the mRNA by a posttranscriptional event (Fig 2C). To discriminate whether the SL sequence should be added by *trans*-splicing or long-range *cis*-splicing, we first determined the transcription start sites (TSSs) of this gene by cap-trapper cDNA sequencing, which revealed their locations at –151, –150, and –136 relative to the ATG codon (Fig 2B, thick arrows). On this basis, we designed primers b and c, which located downstream and upstream of the TSSs, respectively (Fig 2A). A primer pair b–d (Fig 2A) produced PCR fragments of the same size from both genomic and cDNA (Fig 2C), indicating that in our RNA preparations, primer b sequences (primer b consists of two nested primers underlined in Fig 2B) were retained only by the primary transcripts before splicing out the introns. Next, the primer pair c–d gave the amplified fragment only from the genomic DNA but not from cDNA, indicating that this gene does not produce long transcripts starting upstream of the above TSSs. These results reject the possibility that the SL sequence was added to the mRNA by long-range *cis*-splicing, and hence we conclude that SL *trans*-splicing occurs in this organism.

### SL *trans-*splicing occurs for the genes of various metabolism in *P*. *micropora*

**Fig 3:**
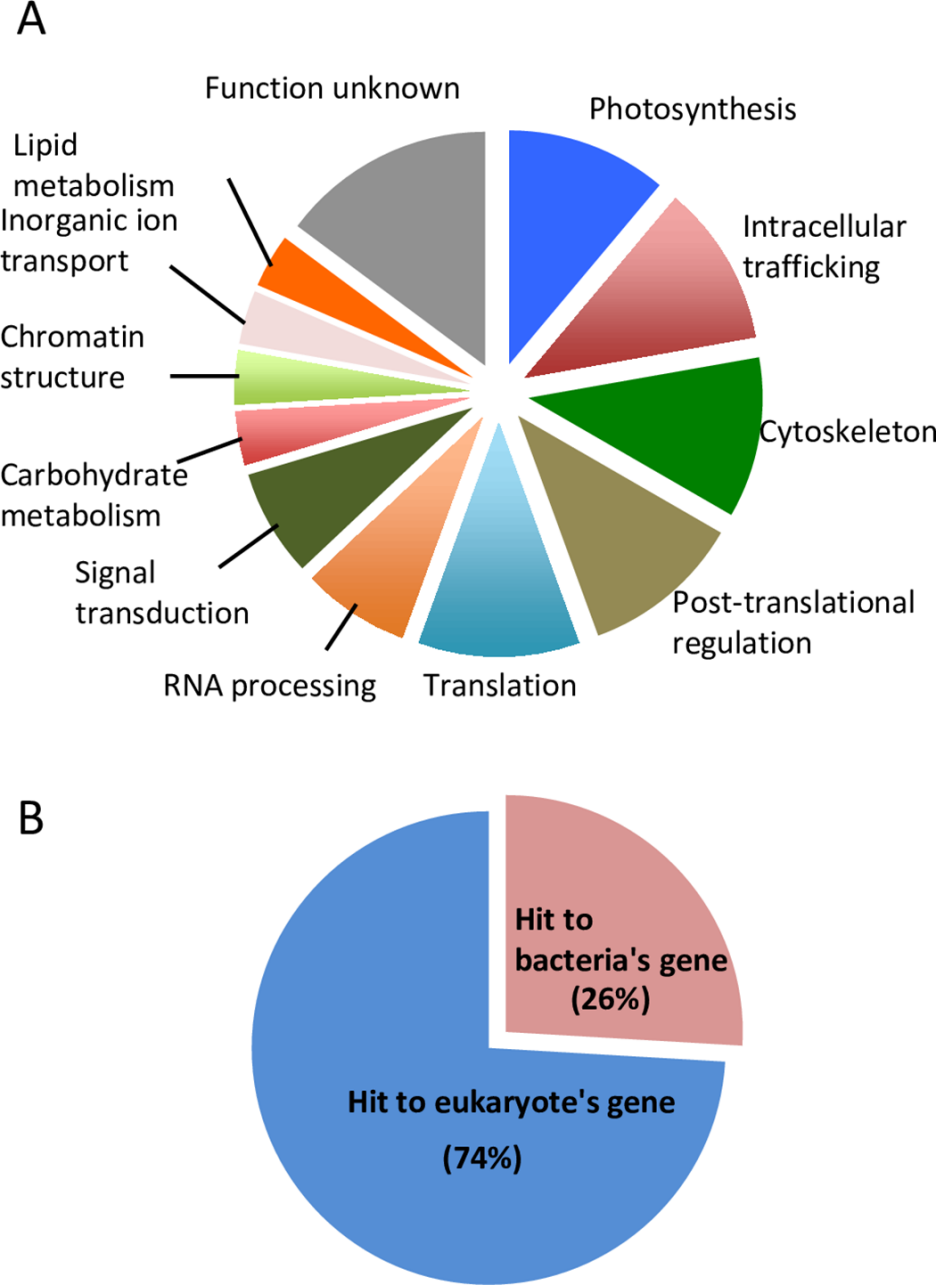
Functional and taxonomical classification of SL *trans*-spliced genes. **A**. Functional categorization of 27 *trans*-spliced genes of *P*. *micropora* according to COG and KOG classification [44, 45] with manual editing; a class of photosynthesis related function is added. **B.** Taxonomical classification of 27 SL *trans*-spliced genes based on the BLASTX annotation of the NCBI-nr database.

When we searched for the SL sequence shown in Fig 1, the cDNA sequence with reads similar to bacterial genes were discarded for reasons mentioned above. However, *P*. *micropora* undoubtedly has its own genes derived from cyanobacterial endosymbiont [39] and probably also from horizontally transferred genes from unidentified bacteria [40]. To salvage those nuclear genes, we collected whole cap-trapper cDNA reads that contain 20-base SL conserved sequence (TGGATAATCCGGCTTTTCTG), and we obtained 1204 reads, which were assembled into 773 contigs. Although these 5′ terminal sequences were generally too short to cover the protein-coding regions, BLASTX analysis detected 11 new protein genes. In conjunction with the genes listed in Fig 1, we detected 27 SL *trans*-spliced genes in total. Their functional classification showed that SL *trans*-splicing in *P*. *micropora* occurs for genes of diverse metabolisms and functions (Fig 3A, S1 Table) similar to the cases of other SL *trans*-splicing organisms [4], and we could not detect any bias for specific cellular functions. Next, we examined whether these 27 genes are similar to either of eukaryotic- or prokaryotic-genes by BLASTX (nr). Interestingly, 7 of them (26%) were assigned to bacterial genes (Fig 3B), which include photosynthetic genes from endosymbiotic cyanobacteria [40]. This indicates that the endosymbiotically and horizontally transferred genes could also be targets of SL *trans*-splicing in this organism.

### Characterization of the SL RNA genes in *P*. *micropora*

**Fig 4:**
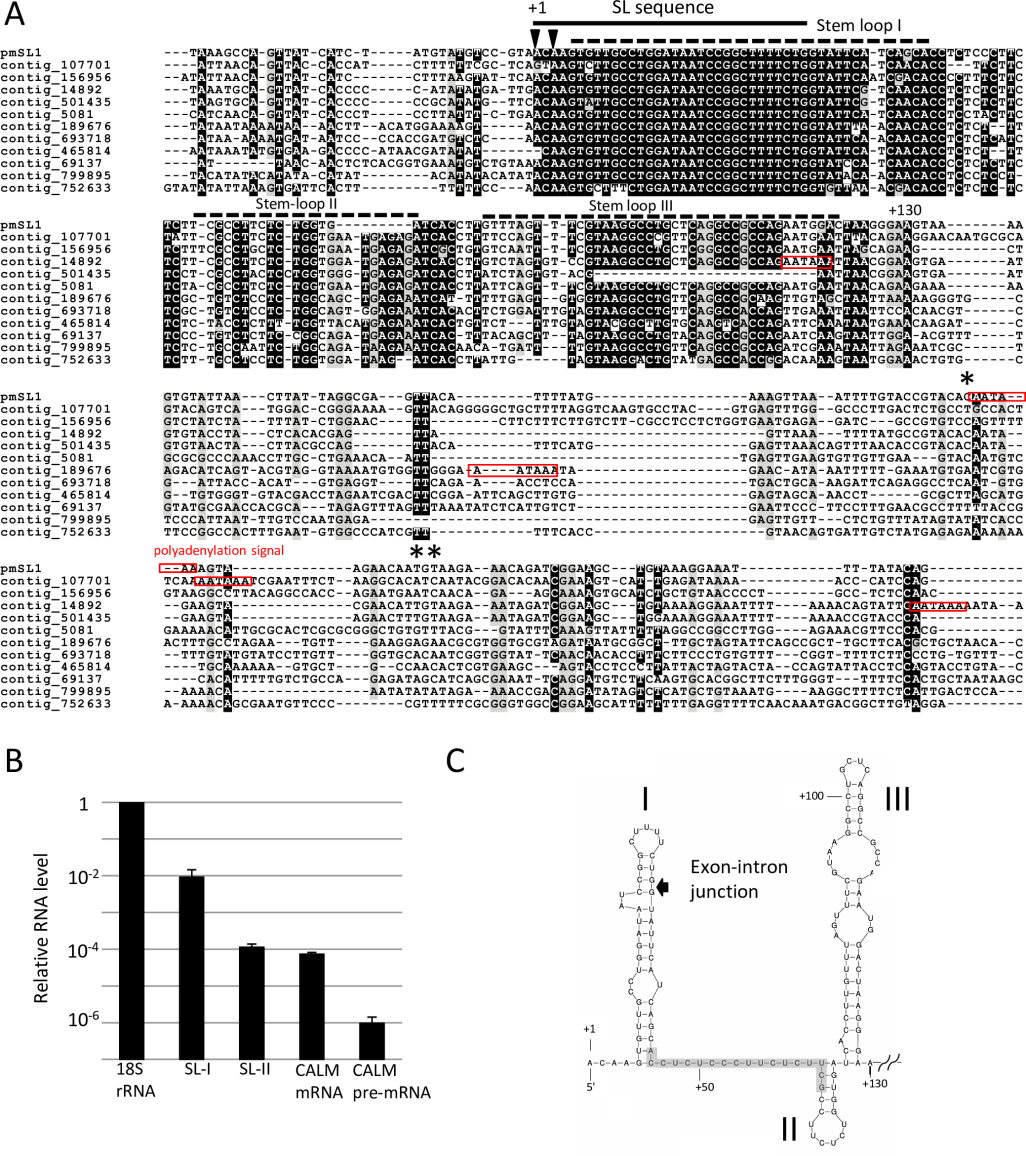
Characteristics of SL RNA genes in *P*. *micropora*. **A.** ClustalW alignment of SL-I RNA gene candidates. pmSL1 represents the Sanger sequence of a PCR-amplified clone, and the others are *de novo* assembled contig sequences from *P*. *micropora* genomic shotgun sequencing. Transcription start sites (arrowheads) of pmSL1 were determined by 5′ end mapping of cap-trapper cDNA reads, and the cleavage sites (asterisks) were determined by 3′ RACE analysis. Canonical poly(A) signals (AATAAA) are indicated by red boxes. The regions forming the stem-loop structure in pmSL1 are represented by dashed lines. **B.** Relative levels of SL-I RNA, SL-II RNA, and calmodulin (CALM) mRNA to 18S rRNA. Bulk expression levels of SL-I and SL-II genes (S2 Fig) were analyzed by real-time RT-PCR using conserved sequences of the respective SL RNA gene group. **C.** Secondary structure of the conserved region of pmSL1 RNA predicted by Mfold [46]. The shaded area indicates the candidate of Sm binding site, when the consensus rule is loosened to pyrimidine rich sequence sandwiched by adenosine and guanosine.

To obtain insight into the SL RNA gene that donates the SL sequence to the pre-mRNAs, we first performed shotgun genome sequencing of *P*. *micropora* MYN1 with Illumina Hiseq 2000 (Illumina, San Diego, CA, USA) and obtained *circa* 438 Mb contig sequences in total (data not shown). We then searched these genomic sequences for the SL RNA genes by a query sequence (TGGATAATCCGGCTTTTCTG-GT), which contained 20-base conserved SL sequence followed by the 5’ splice site motif (GT). Although the genomic contigs were highly fragmented (N50 = 350 bp) because of the large-sized and highly repetitive nature of the *P*. *micropora* genome, we finally detected 62 candidates for the SL RNA genes. They were classified into two subtypes according to their distinct intronic sequences, and we named them SL-I and SL-II (S2 Fig and Fig 4).

Their transcription start sites were determined with the aid of cap-trapper cDNA reads, revealing that the TSSs of SL-1 genes were located at the 5′ terminal region (+1 to +5) of the SL exon, while those of SL-II genes were mainly located at the mid region (+19). This suggests that the whole SL exon sequence was transcribed only from SL-I genes but not from SL-II genes (S2 Fig, arrowheads). Cellular RNA levels of the SL-I and SL-II transcripts were compared with those of the 18S rRNA and with the mature and premature forms of calmodulin mRNAs (Fig 4B). The RNA level of SL-I was about 100 times higher than those of SL-II and calmodulin mature mRNA, and was comparable to 1% of 18S rRNA level. This high-level expression of the SL-1 genes, as well as their TSS positions, indicates that the SL-I genes should be the major source of SL sequences of this organism.

To confirm the above results, which were based on NGS analysis of fragmented sequences, we cloned and Sanger sequenced the genomic and cDNA sequences of an SL-I gene, which we named pmSL1 (Fig 4A). pmSL1 is an ncRNA gene with no long ORFs, and its alignment with the other SL-I gene sequences (S2 Fig) showed that the +1 to +130 base region in pmSL1 is highly conserved, implying the functional importance of this region (Fig 4A). This region is predicted to form a tight RNA secondary structure with three stem-loops (Fig 4C). The splice junction is located near the small bulge within the stem-loop I, representing a typical structure of SL RNA genes [1, 4]. SL RNAs generally encode an Sm-binding site required for SL RNP formation [12, 47, 48]; it is usually located at the single strand region flanking the stem-loops, and its consensus sequence is AAU_3-6_GG/U in trypanosome and nematode [1, 47, 49]. However, we could not find any such typical Sm binding sequence in the conserved region of pmSL1. In *P*. *micropora*, the Sm binding sequence may have diverged from the canonical ones.

To know the 3′ end formation of the pmSL1 RNA, we searched downstream of the above conserved region for the pyrimidine stretch sequence (≥ 6b) containing poly(T) that is used for the transcription termination signal of SL RNA genes in protists [26, 50, 51]. However, we could not find any such pyrimidine stretch; instead, we found a canonical polyadenylation signal, AATAAA, 70 bases downstream of the conserved region (Fig 4A). 3′ RACE analysis showed that pmSL1 RNA was polyadenylated 14 bases downstream of this putative poly(A) signal (Fig 4A, asterisks). In *P*. *micropora*, transcriptional termination of SL RNA genes is likely to occur in a poly(A) signal-dependent manner, which is quite different from that of other SL *trans*-splicing organisms.

## Discussion

This study showed that SL *trans*-splicing occurs in a rhizarian thecate amoeba, the *P*. *micropora* MYN1 strain. Its SL is short (33 bp), contains the UUU triplet, and has G at the 3′ end (Fig 1). This SL *trans*-splicing occurs for genes having diverse cellular functions (Fig 3). In addition, SL RNA can form tight secondary structures (Fig 4). These characteristics of *P*. *micropora's* SL *trans*-splicing represent general features shared by other SL *trans*-splicing organisms known thus far [1, 4]. We found 27 protein-coding genes, most likely SL *trans*-spliced (Fig 1 and S1 Table). However, this number should be the tip of the iceberg, because the number of cap-trapper cDNA reads available in this study was far less than the number covering the total gene (data not shown). To depict the overview of the SL trans-splicing in this organism, more comprehensive analyses of its genome and transcriptome are needed.

Although the *P*. *micropora* SL sequence (33b) shares common structural features with those of other SL *trans*-splicing organisms, it does not show sequence conservation except that of *P*. *chromatophora*: a member of the same photosynthetic *Paulinella* group. This is not surprising, because the *Paulinella* species are phylogenetically distant from other SL *trans*-splicing organisms at the kingdom level [52], and its SL sequence is not conserved at the interphylum level [4]. In nematodes, the SL sequence is involved in the transcriptional regulation of SL RNA genes [53] as well as the translational regulation of *trans*-spliced messages [54]. If this is also the case for *P*. *micropora*, its unique SL sequence may mediate similar functions.

SL *trans*-splicing is observed in two *Paulinella* species, *P*. *micropora* MYN1 (this study) and *P*. *chromatophora* CCAC0185 [27], which are thought to have diverged about 45.7–64.7 million years ago [37, 42]. Therefore, the SL *trans*-splicing ability should have been acquired by the *Paulinella* lineage earlier than their divergence. Notably, SL *trans*-splicing has not been found in other rhizarian organisms: *Bigelowiella natans* [55], *Plasmodiophora brassicae* [56], and *Reticulomyxa filose* [57]. *B*. *natans* and *P*. *brassicae* belong to cercozoa, the same phylum as the *Paulinella* species. Therefore, SL *trans*-splicing should have been acquired uniquely in the *Paulinella* lineage, or multiple independent losses might have occurred in other rhizarian lineages.

In *P*. *micropora*, we found two subtypes of SL RNA genes, namely, SL-I and SL-II, with distinct intronic sequences; we detected 43 SL-I gene copies and 14 SL-II gene copies. Multiple gene copies are general features of SL RNA genes. Because available genome data for *P*. *micropora* were rather incomplete in this study, we expect many more gene copies to be present for SL-I and SL-II. From their expression levels, we expect SL-I genes to be the major source of SL RNAs in this organism, while we do not know whether SL-II genes have specific functions. (Fig 4B).

In this study, we cloned and analyzed an SL-I gene copy, pmSL1, which revealed two intriguing features. One lies in its 3′ end formation. SL RNAs in metazoan, kinetoplastid, and dinoflagellates are usually transcribed as poly(A)-less RNA [2, 3, 25, 58]. A few SL RNAs are exceptionally polyadenylated in dinoflagellates [25] and kinetoplastida [59, 60], with the poly(A) addition occurring at processing sites upstream of the poly(T) tract [50, 59, 60]. In this respect, pmSL1 in *P*. *micropora* is unique in utilizing a canonical polyadenylation signal (AAUAAA) for the addition of poly(A) (Fig 4A). In Fig 4A, 4 of 12 SL-I genes possess the canonical poly(A) signal downstream of the 130-base conserved region, suggesting that at least some portion of the SL-I RNAs are polyadenylated. Poly(T) tract and 3′-box sequence (GTTTAAAACAAGC), found at the transcriptional terminator regions of SL RNA genes in nematode [61], are not found in SL-I RNA genes (Fig 4A). Transcriptional termination and 3′ end formation of SL RNA genes in *P*. *micropora* occurs differently from that of other SL *trans*-splicing organisms.

Another intriguing feature of pmSL1 is the absence of a typical Sm binding sequence (AAU_3-6_GG/U) in the 130-base conserved region. The Sm binding sequence is essential in forming SL RNP and is usually conserved in SL RNAs across the phyla. In relation to this, the Sm binding sequence may have diverged according to Sm protein variants [1, 19, 62]; for example, the Sm binding site of SL RNA (AACUCUCUCCUAUCCCUCUCG) in a tunicate, *Oikopleura dioica*, is far diverged from the canonical sequence [29]. Therefore, it may be possible that *P*. *micropora* has a highly diverged version of the Sm binding sequence. If the consensus sequence rule is relaxed to pyrimidine rich sequence sandwiched by adenine and guanine on the single strand region of SL RNA, a candidate of the Sm binding sequence (ACCUCUCCCUUCUCUUCG) similar to that of *O*. *dioica* is found in the pmSL1 (Fig 4). Further investigation is necessary to elucidate how *P*. *micropora's* SL RNA is involved in SL RNP formation for *trans*-splicing.

This study showed that in *P*. *micropora*, the nuclear genes derived from EGT and HGT are included in the targets of SL *trans*-splicing (Fig 3B). Because this organism is undergoing endosymbiotic evolution [36, 39, 40], it is intriguing to consider whether SL *trans*-splicing can contribute to its evolutionary process. If a given organism undergoes frequent EGT and HGT, its intrinsic genes in the genome might suffer from accidental insertion of the alien gene into the 5’ UTR, which disturbs the translation of the original coding sequence (CDS). This pitfall might be circumvented by SL *trans*-splicing, which directly adds the 5′ cap plus translationally favorable UTR sequence to the original CDS to restore its translatability. From the RNA-seq data of *P*. *micropora*, we found a case that appears to fit our prediction (S4 Fig, S3 Table). In addition, rotifer, an SL *trans*-splicing organism, contains an unusually high number of foreign genes that are acquired horizontally [63, 64]. Therefore, SL *trans*-splicing might be involved in facilitating HGT/EGT. Interestingly, in dinoflagellate, SL *trans-*splicing should have contributed to the amplification and maintenance of the retrotransposed gene, resulting in massive gene birth during adaptive evolution [65]. The facilitative role of SL *trans*-splicing in functional gene transfer and eukaryotic genome evolution deserves further investigation.

In this study, we characterized the SL *trans*-splicing of a photosynthetic thecate amoeba, *Paulinella micropora*, and implied its possible role in HGT/EGT. Further comprehensive analysis of the genome and transcriptome of this organism will provide further insight into the possible contribution of SL *trans*-splicing to endosymbiotic evolution.

## Materials and methods

### Cell culture, RNA- and DNA-extraction

The *P*. *micropora* MYN1 strain was renamed from *P*. *chromatophora* MYN1 [41] on the basis of the morphological trait of *P*. *micropora* [42] and phylogenetic analysis (S3 Fig). The *P*. *micropora* MYN1 strain, which has been deposited at the National Institute for the Environmental Sciences, Tsukuba as NIES-4060, was cultured according to Nomura et al. [41]. The cells were harvested at low speed centrifuge (500 g × 2 min) at 4°C. Total RNAs were extracted using Trizol (Thermo Fisher Scientific) and the total genome was isolated by DNeasy Plant Mini Kit (Qiagen, Hilden, Germany).

### Cap-trapper cDNA library analysis

The *P*. *micropora* cap-trapper cDNA library was constructed according to Carninci and Hayashizaki [43] with slight modifications. 15 μg total RNA sample was used as starting material. Reverse transcription was carried out using the adapter primer plus 15-base random sequence [66]. After cap-trapping, linker ligation and second strand cDNA synthesis were performed according to Shiraki et al. [67]. cDNA products (150–350 bp) were purified by agarose gel electrophoresis and subjected to PCR (32–35 cycles) using linker- and adapter-specific primers. After PCR, 150–350 bp fraction was purified and subjected to the preparation of sequencing libraries with Ion Plus Fragment Library Kit (Thermo Fisher Scientific). Sequencing analyses were performed with Ion PGM^TM^ Sequencer (Thermo Fisher Scientific) (DDBJ Accession No. DRA004751). To determine the TSSs of the calmodulin gene, calmodulin cDNAs were amplified with the linker and calmodulin gene specific primers from the cap-trapper cDNA pool. The linker, adapter, and PCR primer sequences used in this study are described in S2 Table.

### Searching for SL *trans*-spliced gene sequences using cap-trapper cDNA library

Linker- and adapter-sequences of the cap-trapper library were trimmed by Cutadapt software [68], and then short reads (<30b) and reads hitting to rRNA and *P*. *chromatophora's* genes [36] by BLASTN (e-value<1e-10) were discarded. During SL sequence screening, the reads hitting N-terminal 50 amino acid residues of eukaryotic genes in the NCBI-nr database were collected (BLASTX, e-value<1e-5), and then assembled into contigs by CLC Genomic Workbench with default settings (CLC bio, Tokyo, Japan). The contigs’ information and BLASTX results were used to group the cap-trapper reads. The common sequences at the 5′ end of the reads were searched using BLASTN algorithm.

### Genome sequencing, gene structure, and expression analysis

Shotgun genomic sequencing of the *P*. *micropora* genome was performed with Illumina Hiseq2000 (Illumina) (DDBJ Accession No. DRA004743) and assembled by Velvet [69]. The library construction, sequencing, and genome assembly were provided as a customary service by Eurofins MWG Operon LLC (Tokyo, Japan). PCR, RT-PCR, and Sanger sequencing analyses were performed by gene specific primers listed in S2 Table. Gene and cDNA sequences analyzed by Sanger sequencing method are available in DDBJ databank (LC383945, LC383946, LC384061–LC384070). Genome contig sequences associated with SL RNA genes are shown in S3 Table. For gene expression analysis, cDNAs were synthesized with random nonamers and ReverTra Ace (Toyobo Bio-Technology Co., Ltd, Osaka, Japan), then subjected to real-time with Thunderbird SYBR qPCR mix (Tobyobo Bio-Technology, Co., Ltd, Osaka, Japan) and Eco Real-Time PCR System (Illumina). In a 3′ RACE experiment, cDNAs were synthesized using an anchored Oligo (dT)_16_ primer from poly(A)+ RNA, purified with Dynabeads® mRNA DIRECT™ Kit (Thermo Fisher Scientific), and amplified with anchor primer and SL RNA specific primer by PCR. SL RNA structure was predicted by Mfold [46] (also see http://www.bioinfo.rpi.edu/applications/mfold).

## Supporting information

S1 Table COG/KOG classification of SL-containing cDNA reads. (XLSX)

S2 Table Primers and oligonucleotides. (XLSX)

S3 Table Sequences analyzed in Fig 4, S2 Fig and S4 Fig. (XLSX)

S1 Fig Mapping of the cap-trapper cDNA tag-reads on actin and polyubiquitin genes. 5' ends of the tag-reads were mapped by BLASTN (identity ≥ 95%, alignment length/ the read length> 0.9).
(PDF)

S2 Fig SL gene candidates in *P*. *micropora* genome. SL gene candidates detected in the *P*. *micropora* genome contigs by BLASTN analysis using 22bp query sequence, which consists of 20bp SL-conserved sequence (TGGATAATCCGGCTTTTCTG) and 5' splicing motif (GT). The sequences were aligned by MAFFT (ver.7)^#^ and were grouped according to the intron sequences. Exon- and intron-regions of SL-I genes are indicated by solid and dashed bold lines above the sequences. Transcription start sites and their nucleotides, where 5' end of cap trapper cDNA reads are mapped, are shown by arrowheads and red color, respectively. Asterisk means the end of the contig sequence. ^#^ Katoh *et al*. (2002) *Nucleic Acids Res* 30, 3059–3066.
(PDF)

S3 Fig Maximum likelihood tree of 16S rRNA gene sequences of photosynthetic *Paulinella* species. The sequences are analyzed using Kimura-2 +G model by MEGA7 [1] with 1000 bootstrap replication. IDs in parentheses are Genbank- and DDBJ-accessions. *P*. *micropora* FK01 (*) and MYN1 (**), previously reported as *P*. *chromatophora* strains [2,3], were renamed in Lhee et al. [4] and in this study, respectively.
[1] Kumar S, Stecher G, Tamura K. MEGA7: Molecular Evolutionary Genetics Analysis version 7.0 for bigger datasets. Mol. Biol. Evol. 2016; 33: 1870–4.
[2] Yoon HS, Nakayama T, Reyes-Prieto A, Andersen RA, Boo SM, Ishida K, Bhattacharya D. A single origin of the photosynthetic organelle in different *Paulinella* lineages. BMC Evol. Biol. 2009; 9: 98.
[3] Nomura M, Nakayama T, Ishida K. Detailed process of shell construction in the photosynthetic testate amoeba *Paulinella chromatophora* (euglyphid, Rhizaria). J. Eukaryot. Microbiol. 2014; 61: 317–21.
[4] Lhee D, Yang EC, Kim JI, Nakayama T, Zuccarello G, Andersen RA, Yoon HS. Diversity of the Photosynthetic *Paulinella* Species, with the Description of *Paulinella micropora* sp. nov. and the Chromatophore Genome Sequence for strain KR01. Protist. 2017; 168: 155–70.
(PDF)

S4 Fig A case of SL *trans*-spliced mRNA rescued from the disturbance by the retrotransposon insertion just upstream of the coding region. **A.** Schematic models of the premature and SL *trans*-sliced forms of the transcripts encoding a serine/threonine kinase. These RNA sequences were searched from the RNA-seq data of *P*. *micropora* obtained by Illumina HiSeq 4000. **B.** Nucleotide sequences covering the *trans*-splicing site and translation initiation context of the transcripts modeled in **A**, with the 3’ splicing acceptor motif (AG), termination codon, and initiation codon shown by red characters.
(PDF)
