# Supplementary material for: Characterization of spliced leader trans-splicing in a photosynthetic rhizarian amoeba, Paulinella micropora, and its possible role in functional gene transfer
Source: PLoS One. 2018 Jul 19;13(7):e0200961. doi: 10.1371/journal.pone.0200961 (PMC6053224; doi:10.1371/journal.pone.0200961)
Supplement: S1 Fig — 5' ends of the tag-reads were mapped by BLASTN (identity ≥ 95%, alignment length/ the read length> 0.9). (PDF) [file pone.0200961.s004.pdf]

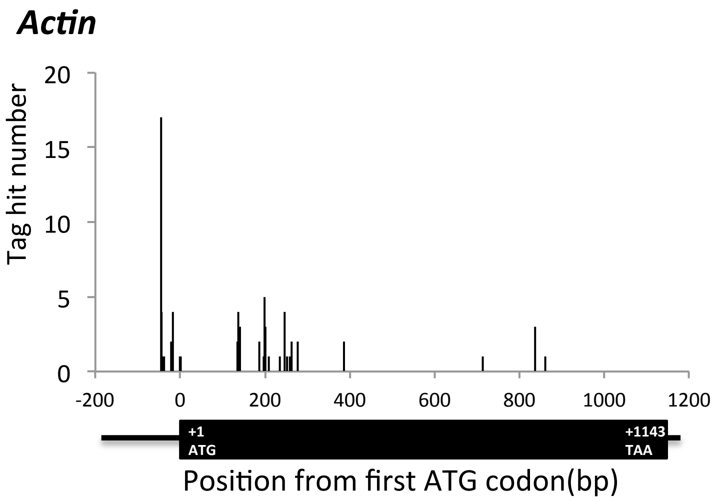

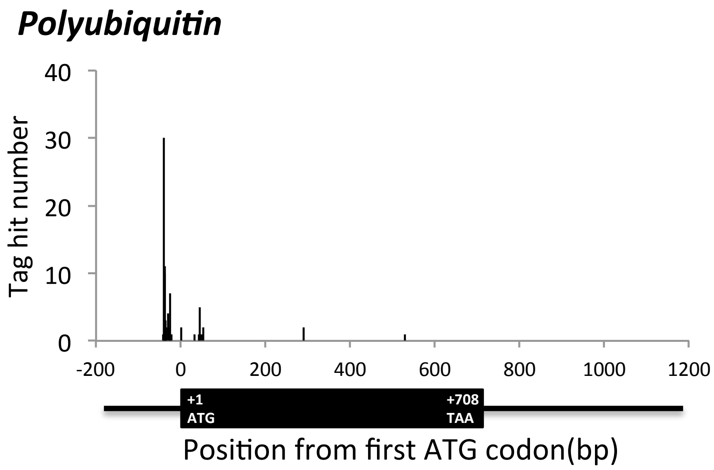

S1 Fig. Mapping of the cap-trapper tag-reads on actin- and polyubiquitin-genes.
5' ends of the tag-reads were mapped by BLASTN (identity ≥ 95%, alignment length/ the read length> 0.9).
