# Supplementary material for: Characterization of spliced leader trans-splicing in a photosynthetic rhizarian amoeba, Paulinella micropora, and its possible role in functional gene transfer
Source: PLoS One. 2018 Jul 19;13(7):e0200961. doi: 10.1371/journal.pone.0200961 (PMC6053224; doi:10.1371/journal.pone.0200961)
Supplement: S2 Fig — SL gene candidates detected in the P. micropora genome contigs by BLASTN analysis using 22bp query sequence, which consists of 20bp SL-conserved sequence (TGGATAATCCGGCTTTTCTG) and 5' splicing motif (GT). The sequences were aligned by MAFFT (ver.7)# and were grouped according to the intron sequences. Exon- and intron-regions of SL-I genes are indicated by solid and dashed bold lines above the sequences. Transcription start sites and their nucleotides, where 5' end of cap trapper cDNA reads are mapped, are shown by arrowheads and red color, respectively. Asterisk means the end of the contig sequence. # Katoh et al. (2002) Nucleic Acids Res 30, 3059–3066. (PDF) [file pone.0200961.s005.pdf]

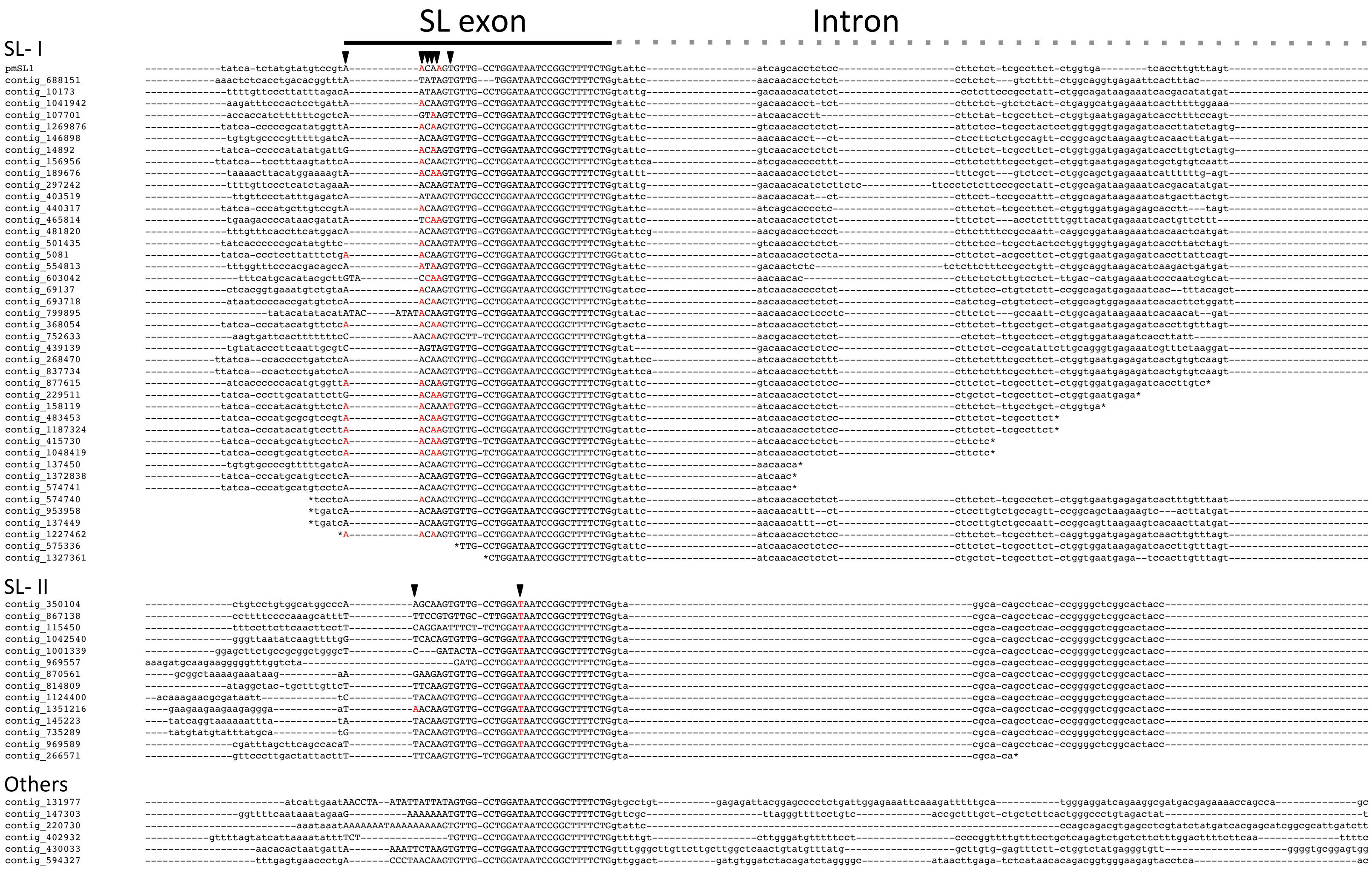

S2 Fig. SL gene candidates in *P. micropora* genome. SL gene candidates detected in the *P. micropora* genome contigs by BLASTN analysis using 22bp query sequence, which consists of 20bp SL-conserved sequence (TGGATAATCCGGCTTTTCTG) and 5' splicing motif (GT). The sequences were aligned by MAFFT (ver. 7)# and were grouped according to the intron sequences. Exon- and intron-regions of SL-I genes are indicated by solid and dashed bold lines above the sequences. Transcription start sites and their nucleotides, where 5' end of cap trapper cDNA reads are mapped, are shown by arrowheads and red color, respectively. Asterisk means the end of the contig sequence. # Katoh *et al.* (2002) *Nucleic Acids Res* 30, 3059-3066.
