# Supplementary material for: Characterization of spliced leader trans-splicing in a photosynthetic rhizarian amoeba, Paulinella micropora, and its possible role in functional gene transfer
Source: PLoS One. 2018 Jul 19;13(7):e0200961. doi: 10.1371/journal.pone.0200961 (PMC6053224; doi:10.1371/journal.pone.0200961)
Supplement: S3 Fig — The sequences are analyzed using Kimura-2 +G model by MEGA7 [1] with 1000 bootstrap replication. IDs in parentheses are Genbank- and DDBJ-accessions. P. micropora FK01 (*) and MYN1 (**), previously reported as P. chromatophora strains [2,3], were renamed in Lhee et al. [4] and in this study, respectively. [1] Kumar S, Stecher G, Tamura K. MEGA7: Molecular Evolutionary Genetics Analysis version 7.0 for bigger datasets. Mol. Biol. Evol. 2016; 33: 1870–4. [2] Yoon HS, Nakayama T, Reyes-Prieto A, Andersen RA, Boo SM, Ishida K, Bhattacharya D. A single origin of the photosynthetic organelle in different Paulinella lineages. BMC Evol. Biol. 2009; 9: 98. [3] Nomura M, Nakayama T, Ishida K. Detailed process of shell construction in the photosynthetic testate amoeba Paulinella chromatophora (euglyphid, Rhizaria). J. Eukaryot. Microbiol. 2014; 61: 317–21. [4] Lhee D, Yang EC, Kim JI, Nakayama T, Zuccarello G, Andersen RA, Yoon HS. Diversity of the Photosynthetic Paulinella Species, with the Description of Paulinella micropora sp. nov. and the Chromatophore Genome Sequence for strain KR01. Protist. 2017; 168: 155–70. (PDF) [file pone.0200961.s006.pdf]

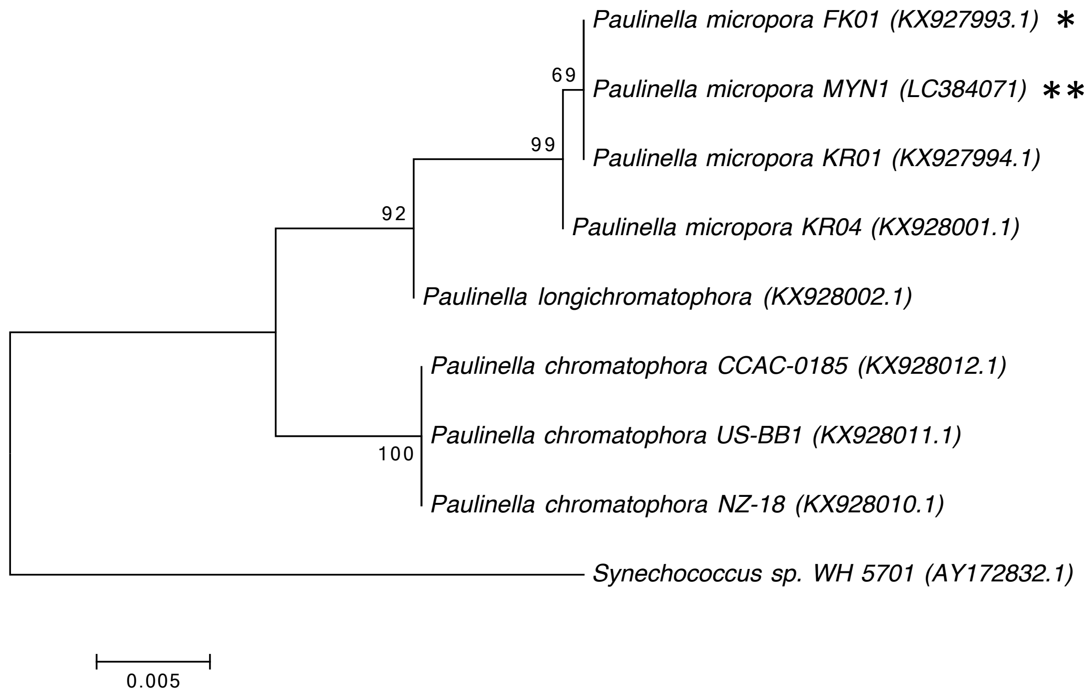

S3 Fig. Maximum likelihood tree of 16S rRNA gene sequences of photosynthetic *Paulinella* species. The sequences are analyzed using Kimura-2 +G model by MEGA7 [1] with 1000 bootstrap replication. IDs in parentheses are Genbank- and DDBJ-accessions. *P. micropora* FK01 (*) and MYN1 (**), previously reported as *P. chromatophora* strains[2,3], were renamed in Lhee *et al*. [4] and in this study, respectively.

[1] Kumar S, Stecher G, Tamura K. MEGA7: Molecular Evolutionary Genetics Analysis version 7.0 for bigger datasets. Mol. Biol. Evol. 2016; 33: 1870-4.

[2] Yoon HS, Nakayama T, Reyes-Prieto A, Andersen RA, Boo SM, Ishida K, Bhattacharya D. A single origin of the photosynthetic organelle in different *Paulinella* lineages. BMC Evol. Biol. 2009; 9: 98.

[3] Nomura M, Nakayama T, Ishida K. Detailed process of shell construction in the photosynthetic testate amoeba *Paulinella chromatophora* (euglyphid, Rhizaria). J. Eukaryot. Microbiol. 2014; 61: 317-21.

[4] Lhee D, Yang EC, Kim JI, Nakayama T, Zuccarello G, Andersen RA, Yoon HS. Diversity of the Photosynthetic *Paulinella* Species, with the Description of *Paulinella micropora* sp. nov. and the Chromatophore Genome Sequence for strain KR01. Protist. 2017; 168: 155-70.
