# Supplementary material for: Characterization of spliced leader trans-splicing in a photosynthetic rhizarian amoeba, Paulinella micropora, and its possible role in functional gene transfer
Source: PLoS One. 2018 Jul 19;13(7):e0200961. doi: 10.1371/journal.pone.0200961 (PMC6053224; doi:10.1371/journal.pone.0200961)
Supplement: S4 Fig — A. Schematic models of the premature and SL trans-sliced forms of the transcripts encoding a serine/threonine kinase. These RNA sequences were searched from the RNA-seq data of P. micropora obtained by Illumina HiSeq 4000. B. Nucleotide sequences covering the trans-splicing site and translation initiation context of the transcripts modeled in A, with the 3’ splicing acceptor motif (AG), termination codon, and initiation codon shown by red characters. (PDF) [file pone.0200961.s007.pdf]

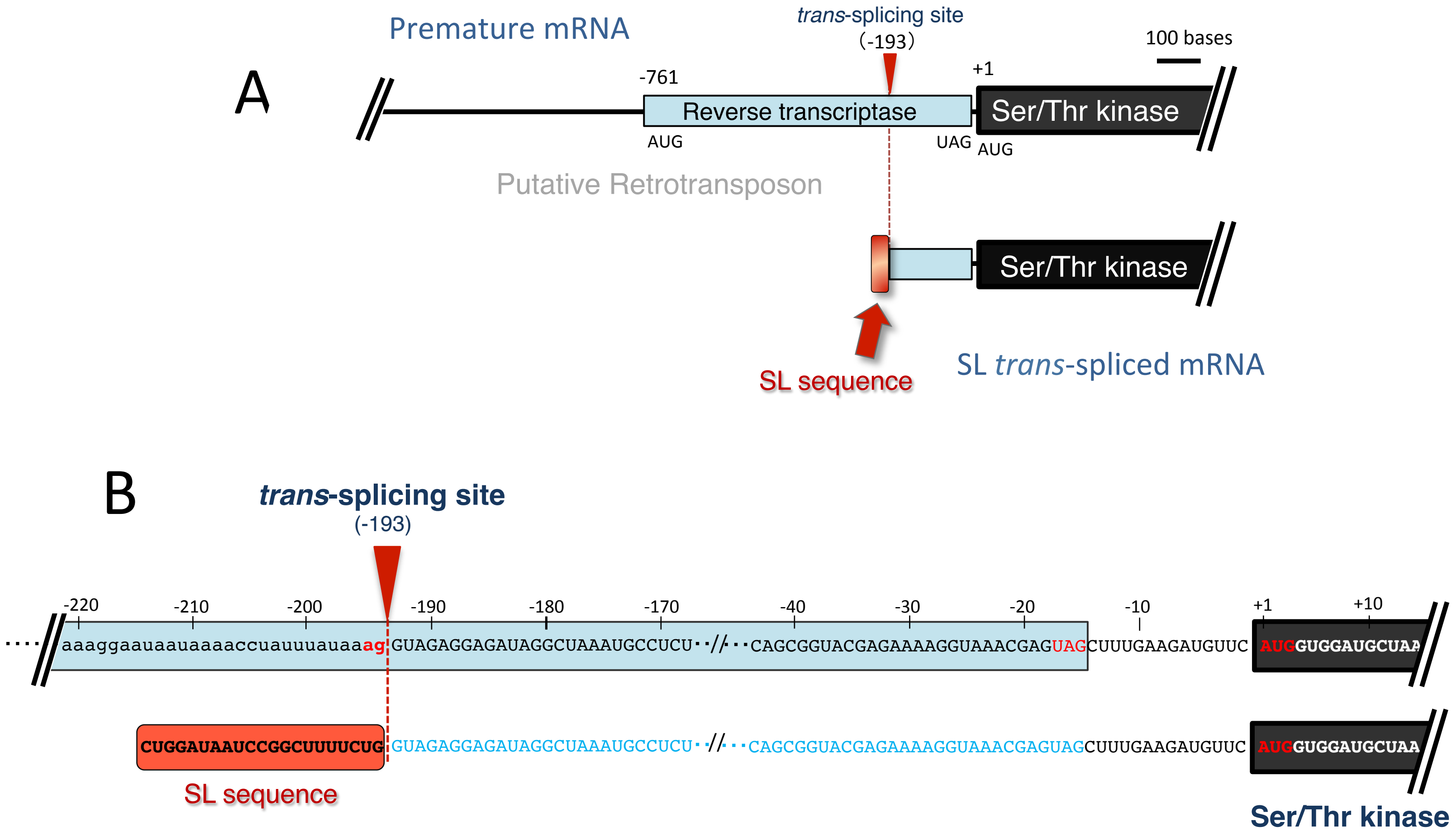

S4 Fig. A case of SL *trans*-spliced mRNA rescued from the disturbance by the retrotransposon insertion just upstream of the coding region. A. Schematic models of the premature and SL *trans*-sliced forms of the transcripts encoding a serine/threonine kinase. These RNA sequences were searched from the RNA-seq data of *P. micropora* obtained by Illumina HiSeq 4000. **B.** Nucleotide sequences covering the *trans*-splicing site and translation initiation context of the transcripts modeled in **A**, with the 3' splicing acceptor motif (AG), termination codon, and initiation codon shown by red characters.
